# Supplementary material for: NOD-Like Receptor Genes Undergo Diversity-Enhancing Evolution in a Fungal Species Complex
Source: Genome Biol Evol. 2026 Jul 23;18(8):evag185. doi: 10.1093/gbe/evag185 (PMC13449251; doi:10.1093/gbe/evag185)
Supplement: Supplementary file 1 [file evag185_supplementary_data.zip › MolEvoNLRs_GBE_Suppl_Final.pdf]

Supplementary Materials of:

NOD-like receptor genes evolve under diversity-enhancing evolution in a fungal species complex

S. Lorena Ament-Velásquez, Sven J. Saupe

Methods

Gene searches and annotation

Previous studies detected proteins related to the *hwnd* genes by doing BLASTp (Altschul *et al.*, 1997) searches with a family member, in particular HET-E. This led to the discovery of the *nwd1*, *nwd2*, *nwd3*, *nwd4*, and *nwd5*, as well as the pseudogenes *nwdp-1* (here *nwd6*) and *nwdp-2*. To find more homologs, we performed similar searches on the NCBI website (https://blast.ncbi.nlm.nih.gov/Blast.cgi) while fixing the taxid to *P. anserina* (2587412). However, the BLASTp searches depend on good gene models that are often of bad quality for STAND proteins (Yuen, Bayes and Degnan, 2014). Hence, we performed tBLASTn searches with the NACHT domain protein sequence of HET-E (accession number XP_001903815.1, residues 294 to 738), as well as NWD4 (accession number CDP29667.1, residues 339 to 759), which has the most divergent NACHT domain relative to *het-e* from the known NWD genes. As reference, we used the Podan2 assembly from the S+ strain (Espagne et al. 2008), available on the Joint Genome Institute MycoCosm website (https://mycocosm.jgi.doe.gov/Podan2/Podan2.home.html). The tBLASTn searches were performed with the script query2haplotype.py v. 2.00 (https://github.com/SLAment/Genomics/blob/master/BLAST/query2haplotype.py) with options --

haplo --task tblastn -e 0.1, which fuses overlapping BLAST hits and defines individual haplotypes based on hits that are not farther than 10 kb from each other (controlled with the --vicinity option). We only kept resulting haplotypes that overlapped with at least one gene model from Podan2 and that had a recognizable core NACHT domain, including the ATP/GTPase P-loop (Walker A) motif, composed of the sequence GxxxxGK[ST], and the Mg2+-binding site (Walker B) motif (Walker *et al.*, 1982; Koonin and Aravind, 2000).

Similarly, we used the NB-ARC domain of PaPLP1 (accession number CDP23496.1, positions 371 to 672) as query to search for related NLRs with the same methodology as above. We confirmed that the NB-ARC domain contains the P-loop motif, composed of the sequence GxxGxGK[ST] (van der Biezen and Jones, 1998). The gene *Pa_1_5560* was excluded from the results as it is likely pseudogenized in all species and there was no clear evidence to define the correct gene model. Additional genes were included if the NB-ARC domain (Pfam PF00931) was detected by InterProScan v. 5.62-94.0 (Jones *et al.*, 2014) with default parameters.

We found multiple inconsistencies between the Podan2 gene models, more updated gene models of the S+ reference (Lelandais et al. 2022), an alternative annotation pipeline termed nice-3.00 (Ament-Velásquez et al. 2024), and the splicing sites suggested by RNAseq data of *P. anserina* (sample Psk2xS14-vsS mapped to the Podan2 assembly) or *P. comata* (samples PcTdp and PcWa131m mapped to the PcWa139m assembly) produced previously (Vogan *et al.*, 2019). We gave priority to the RNAseq data patterns to choose or correct gene models amongst all the options when necessary. In the absence of RNAseq expression (<10x) in both *P. anserina* and *P. comata*, comparisons were made with the closest BLASTp hit in NCBI, which usually came from the genomes of *Apiosordaria backusii* strain CBS 540.89 and *Podospora setosa* strain CBS 892.96. Otherwise, the Podan2 gene model was taken as the correct one. Most of the inconsistencies were due to stop-codons, frame-shifts, or transposon insertions present in the S+

genome, which were often not there in homologs from other species detected by BLASTp in NCBI (last consulted on 2024-03-25). Hence, we marked the genes as potential pseudogenes. Occasionally, a given NLR was fragmented into two gene models in Podan2 due to such stop codons, frame-shifts, or transposons. In those cases, the final gene model was renamed by fusing their IDs (e.g., *Pa_6_2410+Pa_6_2405*). After gene-model corrections, we kept a total of 41 NWD-related sequences (including the outgroup Pa_6_7270) and 13 NB-ARC NLRs for downstream analysis, although we detected at least 12 more pseudogenized remains of NWD relatives along the S+ genome that did not fulfill our NACHT minimum requirements.

Gene functional annotation was performed by inputting the corrected protein sequences to the NCBI Conserved Domains Database (Wang *et al.*, 2023), as well as the hmmscan search engine (protein sequence vs profile-HMM database) at the HmmerWeb v. 2.41.2 (https://www.ebi.ac.uk/Tools/hmmer/search/hmmscan), with all "Protein Families" selected and default parameters (E-value = 0.01 and Hit = 0.03). Non-significant annotations were considered if they were consistent with known N-terminus domains in fungal STAND proteins (Dyrka *et al.*, 2014). Overlapping annotations were merged for some Pfam categories with multiple variants (e.g., ANK repeats) following Dyrka et al. (2014) (**Table S1**). Classification of SesA and SesB sequences was further confirmed by comparing sequence alignments to the expected conserved motifs (Graziani, Silar and Daboussi, 2004).

To characterize the evolution of the NLRs at shallow evolutionary times, we retrieved the ortholog of each gene in all the *P. anserina* strains with long-read assemblies, as well as all available assemblies from the other six known species of the *P. anserina* species complex (**Table S3**). In this dataset, the genome of at least one representative of all species has been sequenced with long-reads (only one strain is available for *P. bellae-mahoneyi* and *P. pseudopauciseta*). We used the CDS of the reference Podan2 annotation (strain S+) as a query for the `query2haplotype.py`

script to retrieve the nucleotide sequence of each gene, and confirmed orthology by examining the identity of the flanking genes. For every species, we classified the status of each gene as present, absent, pseudogenized, polymorphic, or moved. We inferred the movement (translocation) of genes based on comparisons of synteny of flanking genes, assuming that the chromosomal location that is most common amongst the seven species represents the ancestral state. We considered a gene pseudogenized if we observed frame-shifts, stop codons, or transposon insertions in the coding exons for most of the gene body except the last 50 amino acids of the C-terminus, under the assumption that changes in the position of the stop codon might still produce a fully functional protein. In the case of NLRs with HIC in the C-term domain, any frame-shifts or stop codons in the repeats themselves were ignored as the genes could be "resurrected" by repeat exchange. Loss of start codons was not considered a cause of pseudogenization if another methionine was located within 20 adjacent amino acids. Occasionally, some gene models would have a first exon extending farther away from the RNA-seq reads mapping area. In those cases, we looked for the next methionine downstream of the original start codon that was fully covered by RNA-seq reads and allowed stop-codons or frame-shifts to occur in the upstream exon region that lacked RNA-seq coverage (i.e., they were not marked as pseudogenes if mutations were present). As all strains with long-read assemblies of wildtype strains in our dataset have also been sequenced with Illumina Technology (Vogan *et al.*, 2019, 2021; Hartmann *et al.*, 2021; Ament-Velásquez *et al.*, 2022), we verified all frame-shifts in SPAdes v. 3.12.0 (Bankevich *et al.*, 2012) assemblies built with k-mers 21,33,55,77. Discordance between long-read and Illumina assemblies was typically located in runs of homopolymers, a known issue with these technologies (Delahaye and Nicolas, 2021). In the case of samples that only had Illumina data available, the assembly often had tracks of Ns (missing data) within the repeat domain with HIC or was frequently fragmented into two or more contigs, making it impossible to assess mutations there. The alleles of the *nwd2* and *het-s* genes in the Wageningen Collection were extracted from SPades assemblies produced in the same way.

To contrast the dynamics of the NLR genes with other types of genes in the genome, we randomly selected 100 genes with CDS at least 1 kb-long (NWD-like family median size: 3.4 kb) from the Podan2 annotation and annotated them in the same way as above. For each gene, three sources of gene models were evaluated against the RNA-seq data: the Podan2 and nice-3.00 annotations in *P. anserina*, as well as the equivalent annotation in the reference assembly of *P. comata* (strain Td+, assembly PODCO) (Silar *et al.*, 2019). The gene model of Pa_5_340 was ambiguous but we confirmed that the N-terminus domain is truncated by comparing its AlphaFold model to close homologs (e.g., KAK0659194.1) and considered it a pseudogene. In the case of Pa_7_10370, the stop codon in the Podan2 and nice-3.0 models locates within the ANK domain, while other species have a stop codon downstream outside any HIC repeat. An earlier stop codon in *P. anserina* is also present in an ANK repeat but within a small intron that is not supported by RNA-seq data. While this implies pseudogenization, the RNA-seq expression is considerable (~100x) and decays after the first stop codon. If the gene of a given strain was not fully assembled or the read mapping was not conclusive, it was considered missing data (e.g., Pa_7_11560 in Wa131-, Pa_3_4260 in Wa53-, and Pa_7_11300 in CBS333.63+).

References

Altschul, S.F. *et al.* (1997) "Gapped BLAST and PSI-BLAST: a new generation of protein database search programs," 25(17), pp. 3389–3402.

Ament-Velásquez, S.L. *et al.* (2022) "Allorecognition genes drive reproductive isolation in *Podospora anserina*," *Nature Ecology & Evolution*, 6, pp. 910–923. Available at: https://doi.org/10.1038/s41559-022-01734-x

Ament-Velásquez, S.L. *et al.* (2024) "High-Quality Genome Assemblies of 4 Members of the *Podospora anserina* Species Complex," *Genome Biology and Evolution*, 16(3), p. evae034. Available at: https://doi.org/10.1093/gbe/evae034

Bankevich, A. *et al.* (2012) "SPAdes: a new genome assembly algorithm and its applications to single-cell sequencing," *Journal of Computational Biology*, 19(5), pp. 455–77. Available at:

https://doi.org/10.1089/cmb.2012.0021

van der Biezen, E.A. and Jones, J.D. (1998) “The NB-ARC domain: a novel signalling motif shared by plant resistance gene products and regulators of cell death in animals.,” *Current biology: CB*, 8(7), pp. 225–227. Available at: https://doi.org/10.1016/s0960-9822(98)70145-9

Delahaye, C. and Nicolas, J. (2021) “Sequencing DNA with nanopores: Troubles and biases,” *PLOS ONE*, 16(10), p. e0257521. Available at: https://doi.org/10.1371/journal.pone.0257521

Dyrka, W. *et al.* (2014) “Diversity and variability of NOD-like receptors in fungi,” *Genome Biology and Evolution*, 6(12), pp. 3137–3158. Available at: https://doi.org/10.1093/gbe/evu251

Graziani, S., Silar, P. and Daboussi, M.-J. (2004) “Bistability and hysteresis of the ‘Secteur’ differentiation are controlled by a two-gene locus in *Nectria haematococca*,” *BMC Biology*, 2(1), p. 18. Available at: https://doi.org/10.1186/1741-7007-2-18

Hartmann, F.E. *et al.* (2021) “Size Variation of the Nonrecombining Region on the Mating-Type Chromosomes in the Fungal *Podospora anserina* Species Complex,” *Molecular Biology and Evolution*, 38(6), pp. 2475–2492. Available at: https://doi.org/10.1093/molbev/msab040

Jones, P. *et al.* (2014) “InterProScan 5: genome-scale protein function classification,” *Bioinformatics*, 30(9), pp. 1236–1240. Available at: https://doi.org/10.1093/bioinformatics/btu031

Koonin, E.V. and Aravind, L. (2000) “The NACHT family - A new group of predicted NTPases implicated in apoptosis and MHC transcription activation,” *Trends in Biochemical Sciences*, 25(5), pp. 223–224. Available at: https://doi.org/10.1016/S0968-0004(00)01577-2

Silar, P. *et al.* (2019) “A gene graveyard in the genome of the fungus *Podospora comata*,” *Molecular Genetics and Genomics*, 294(1), pp. 177–190. Available at: https://doi.org/10.1007/s00438-018-1497-3

Vogan, A.A. *et al.* (2019) “Combinations of *Spok* genes create multiple meiotic drivers in *Podospora*,” *eLife*, 8, p. e46454. Available at: https://doi.org/10.7554/eLife.46454

Vogan, A.A. *et al.* (2021) “The Enterprise, a massive transposon carrying *Spok* meiotic drive genes,” *Genome Research*, 31(5), pp. 789–798. Available at: https://doi.org/10.1101/gr.267609.120

Walker, J.E. *et al.* (1982) “Distantly related sequences in the alpha- and beta-subunits of ATP synthase, myosin, kinases and other ATP-requiring enzymes and a common nucleotide binding fold.,” *The EMBO Journal*, 1(8), pp. 945–951. Available at: https://doi.org/10.1002/j.1460-2075.1982.tb01276.x

Wang, J. *et al.* (2023) “The conserved domain database in 2023,” *Nucleic Acids Research*, 51(D1), pp. D384–D388. Available at: https://doi.org/10.1093/nar/gkac1096

Yuen, B., Bayes, J.M. and Degnan, S.M. (2014) “The characterization of sponge NLRs provides insight into the origin and evolution of this innate immune gene family in animals,” *Molecular Biology and Evolution*, 31(1), pp. 106–120. Available at: https://doi.org/10.1093/molbev/mst174

Supplementary Figures

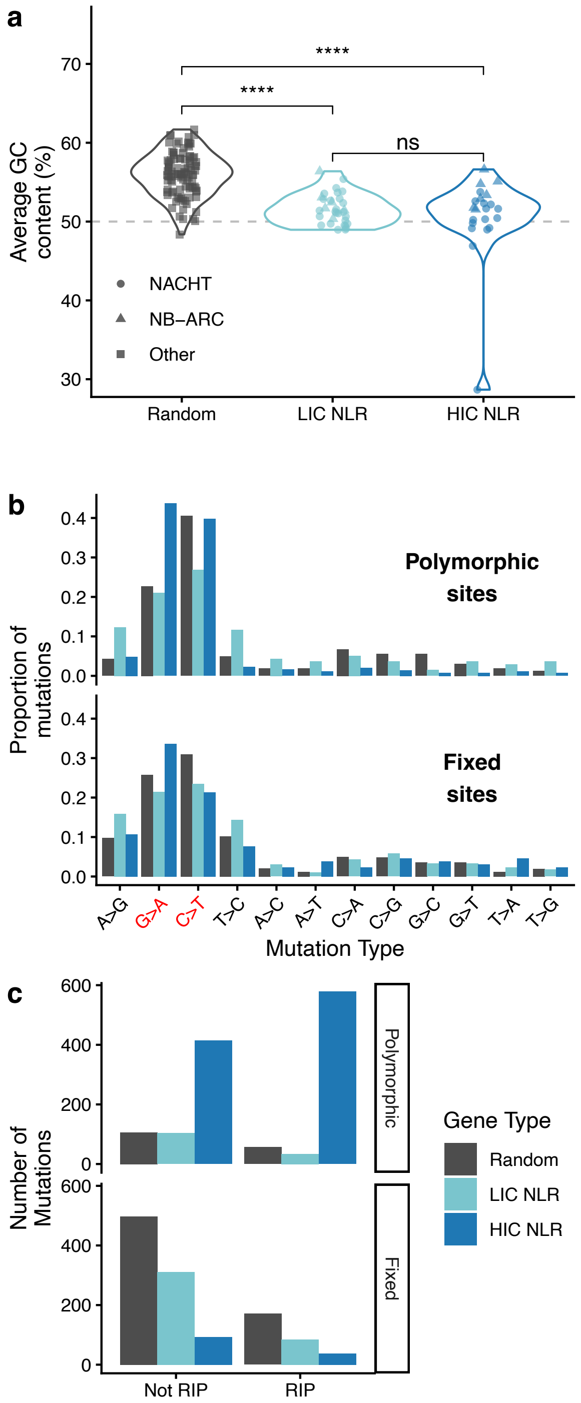

Figure S1. Effect of repeat-induced point mutation (RIP) on *P. anserina* genes. (**a**) Average GC content across gene classes. Significance determined with Kruskal-Wallis rank sum tests followed by a *post hoc* Dunn's test with Bonferroni correction (****: $p <=$ 0.0001; ns: not significantly different). (**b**) Proportion of mutations, polymorphic or fixed within *P. anserina*, occurring in random genes, normal NLRs, or NLRs with HIC. The two transition types associated with RIP are highlighted in red. Mutation types are ordered such that transitions are first, followed by transversions. (**c**) Counts of mutations classified as either RIP-like (C>T mutation proceeded by an A or T, or G>A mutation preceeded by an A or T) or not RIP-like across gene types.

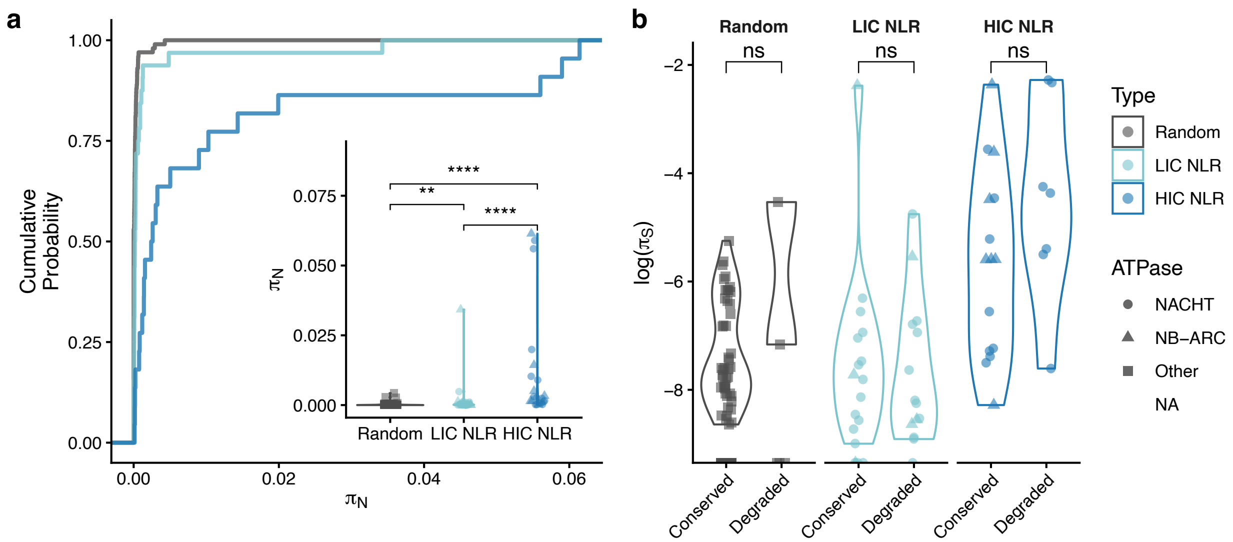

Figure S2. Diversity metrics in *P. anserina* genes. (**a**) Empirical cumulative distribution (ECDF) plot of pairwise-nucleotide non-synonymous diversity (π_N) for different gene classes, with distributions in a violin plot as inset. (**b**) Distribution of pairwise-nucleotide synonymous diversity (π_S) for different gene classes. Genes were further divided into "conserved" (putatively functional for all strains) and "degraded" (pseudogenized in all or some strains). Significance determined by Wilcoxon rank sum tests (ns: not significantly different).

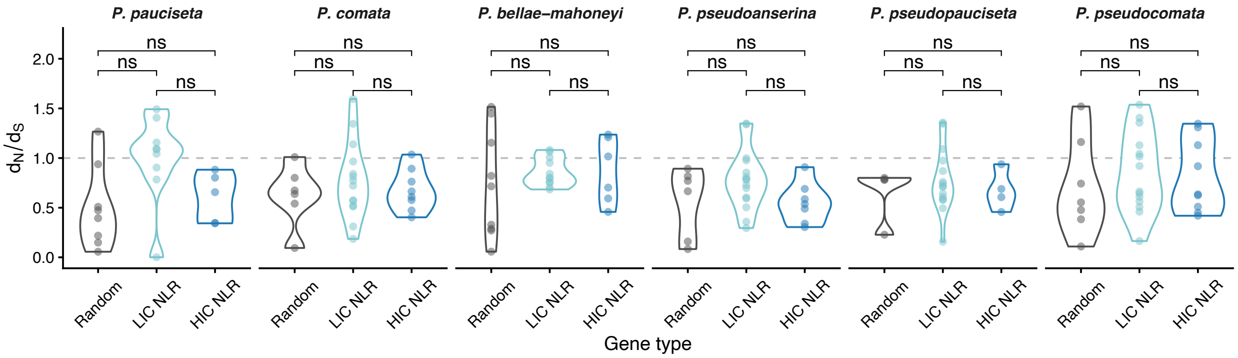

Figure S3. Distribution of d_N/d_S values between *P. anserina* (strain S+) and the ortholog of the other species (type strains) for degraded genes. The dashed gray line marks $d_N = d_S$. Significance determined by Wilcoxon rank sum tests (ns: not significantly different).

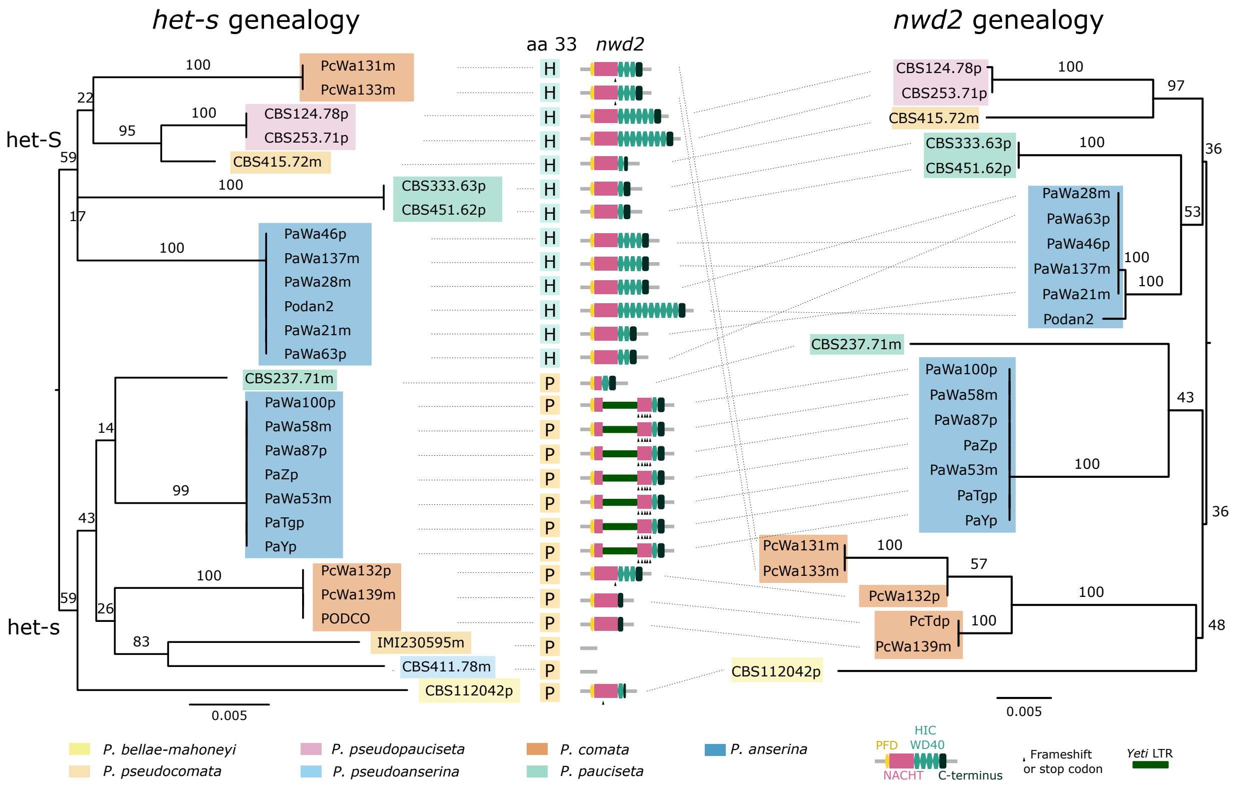

Figure S4. Phylogenetic relationships of *het-s* and *nwd2* in the *Podospora* species. To the left, a maximum likelihood phylogeny of the *het-s* gene shows a split that matches the two alleles, which are defined by the presence of a proline (allele het-s) or a histidine (allele het-S) in the protein position 33. In the center of the figure, the corresponding position 33 of HET-s/S is illustrated along with a cartoon of the domain architecture of the linked *nwd2* allele. To the right, a maximum likelihood phylogeny of *nwd2* partially mirrors that of *het-s*. The exact number of WD40 repeats might be inaccurate for samples without long-read sequencing data. Branch support values correspond to standard non-parametric bootstrap (low within-species values are omitted for clarity). Branches are shown to scale as indicated by the scale bar (nucleotide substitutions per site). PFD: prion forming domain; NACHT: domain present in NAIO, CIITA, HET-E, TP-1; HIC WD40: repeats of the WD40 type with high internal conservation.

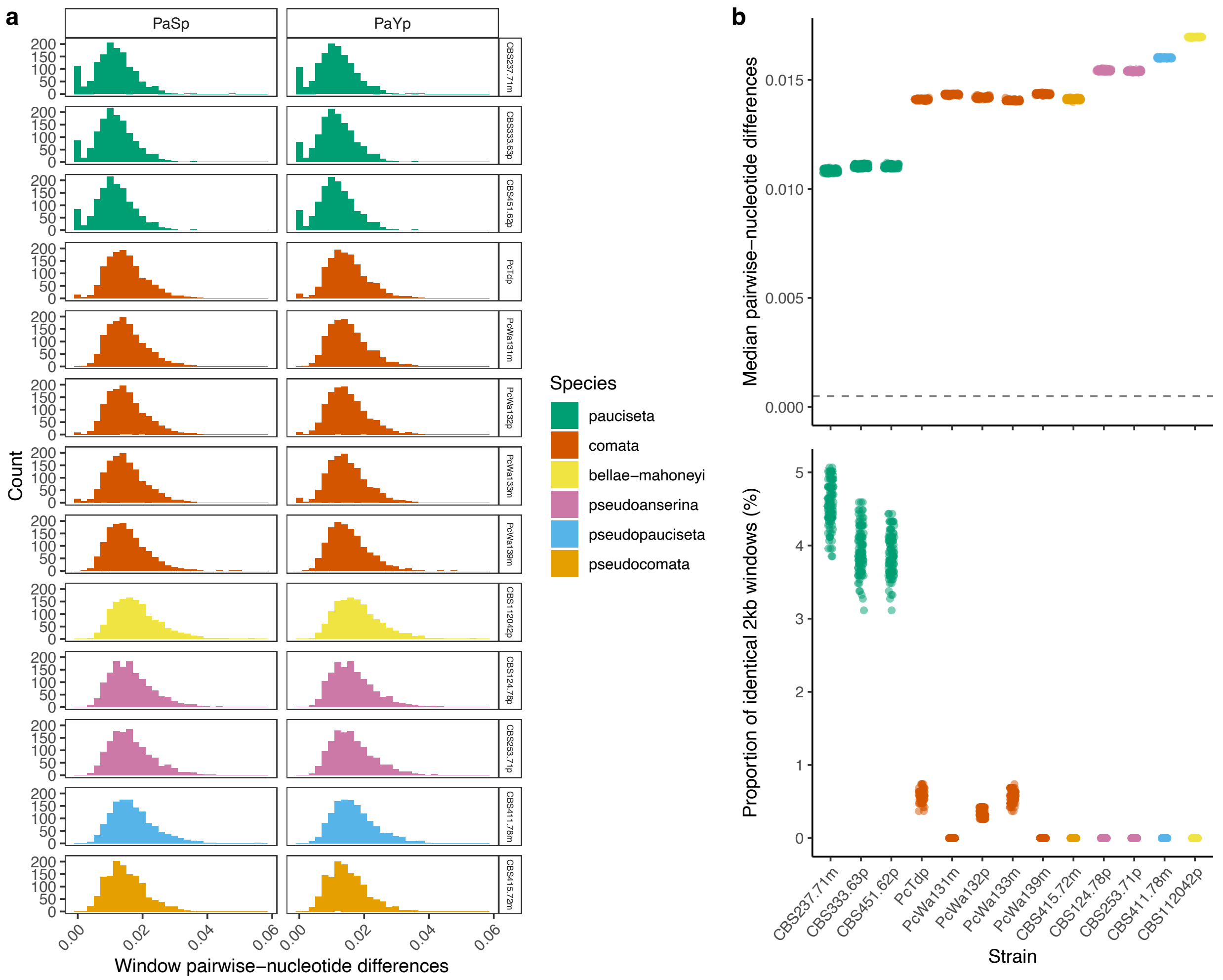

Figure S5. Distribution of divergence between *Podospora* species in chromosome 4. The pairwise-nucleotide differences (divergence) in 2 kb non-overlapping windows were calculated between two representative *P. anserina* strains (PaSp and PaYp) and each of the available strains of the other six species. In (**a**) the distribution of divergence values shows an excess of low divergence windows in comparisons with *P. pauciseta* and, to a lesser degree, some *P. comata* strains. The median window divergence values is very homogenous for all 106 *P. anserina* strains (**b**, top). However, the *Podospora* spp. strains have different proportions of nearly identical windows (divergence <= *P. anserina*'s π) relative to the 106 *P. anserina* strains, represented by individual data points (**b**, bottom). The dashed gray line marks the value of *P. anserina*'s π.

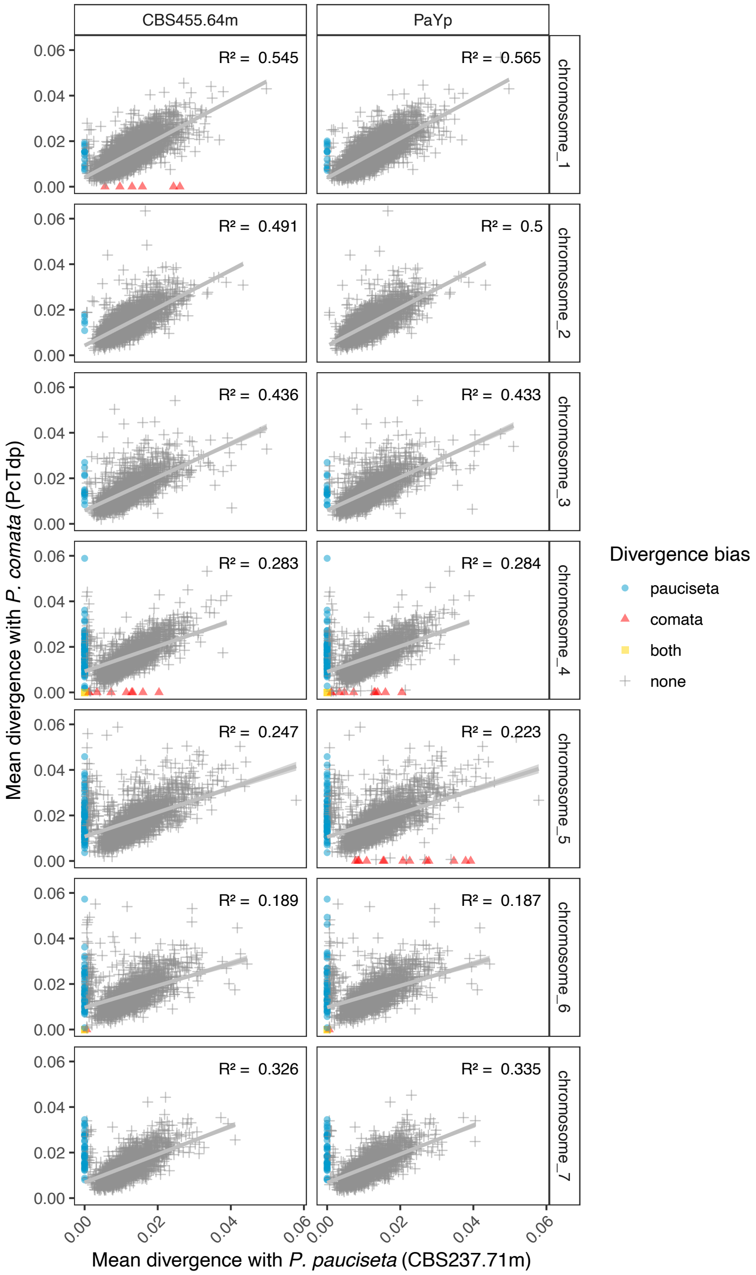

Figure S6. The divergence between *P. anserina* and *P. pauciseta* is highly correlated with the divergence between *P. anserina* and *P. comata*. Comparisons are shown for two example *P. anserina* strains (PaYp and CBS455.64m) against the type strain of either *P. pauciseta* (CBS237.71m) or *P. comata* (PcTdp). Individual points correspond to nucleotide differences (divergence) in 2 kb non-overlapping windows (maximum 1 kb missing data), n = 14814 windows. The windows with divergence values equal or smaller than *P. anserina*'s intra-species diversity (π = 0.000497) are highlighted for either species or both. The R^2 values correspond to linear regressions.

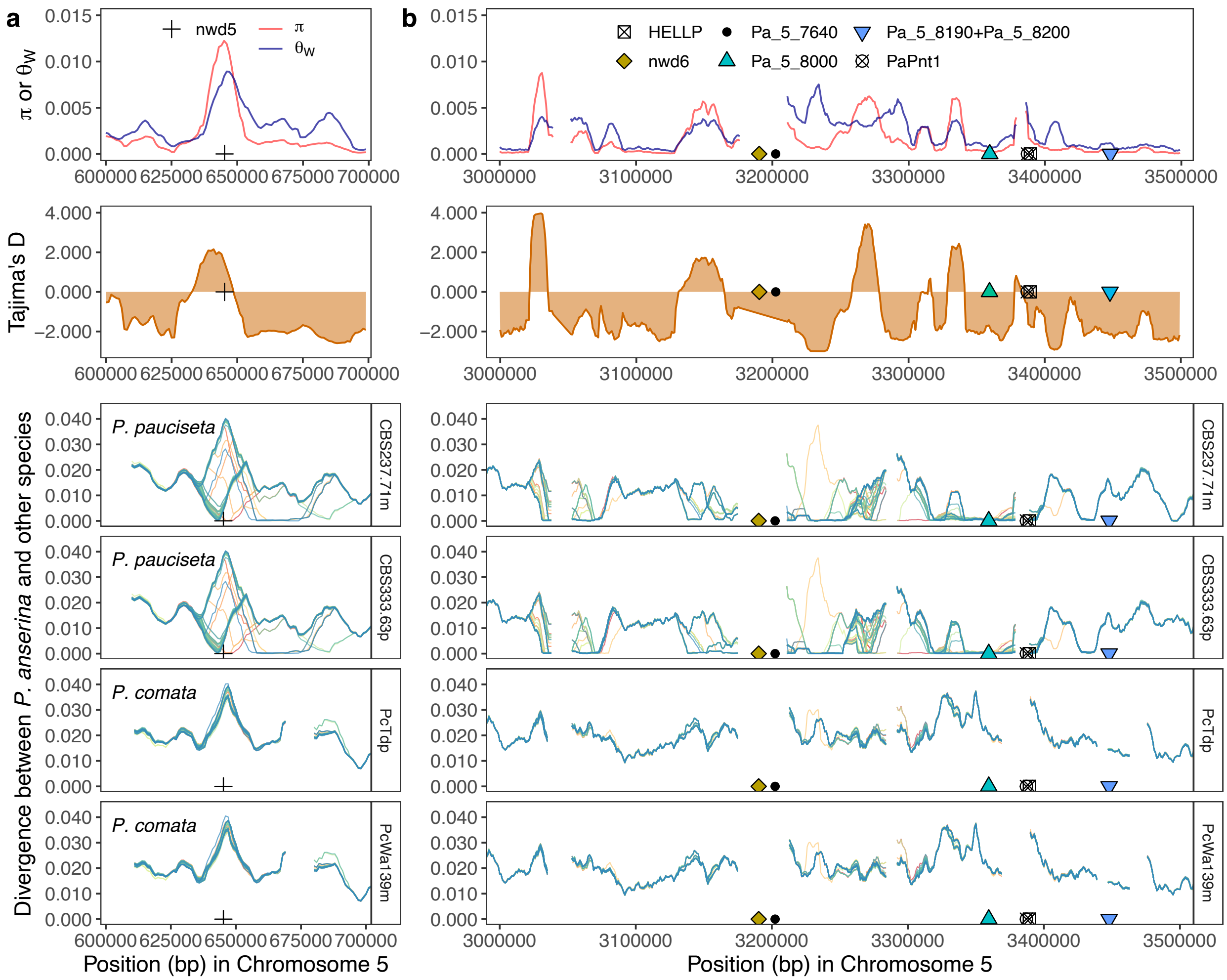

Figure S7. Population diversity and sequence divergence of regions containing NLRs in chromosome 5. The top panels correspond to the two metrics of genetic diversity π and Watterson's θ as well as Tajima's *D* in 10 kb windows (steps of 1 kb), as estimated in Ament-Velásquez et al. (2022) for the Wageningen population of *P. anserina*. The lower panels compare the pairwise-divergence between all 106 *P. anserina* strains vs. two different strains of either *P. pauciseta* and *P. comata*. Each *P. anserina* strain line was assigned a different color (gaps correspond to missing data). (a) Close-up to the *nwd5* region. (b) Larger complex region with several NLRs.

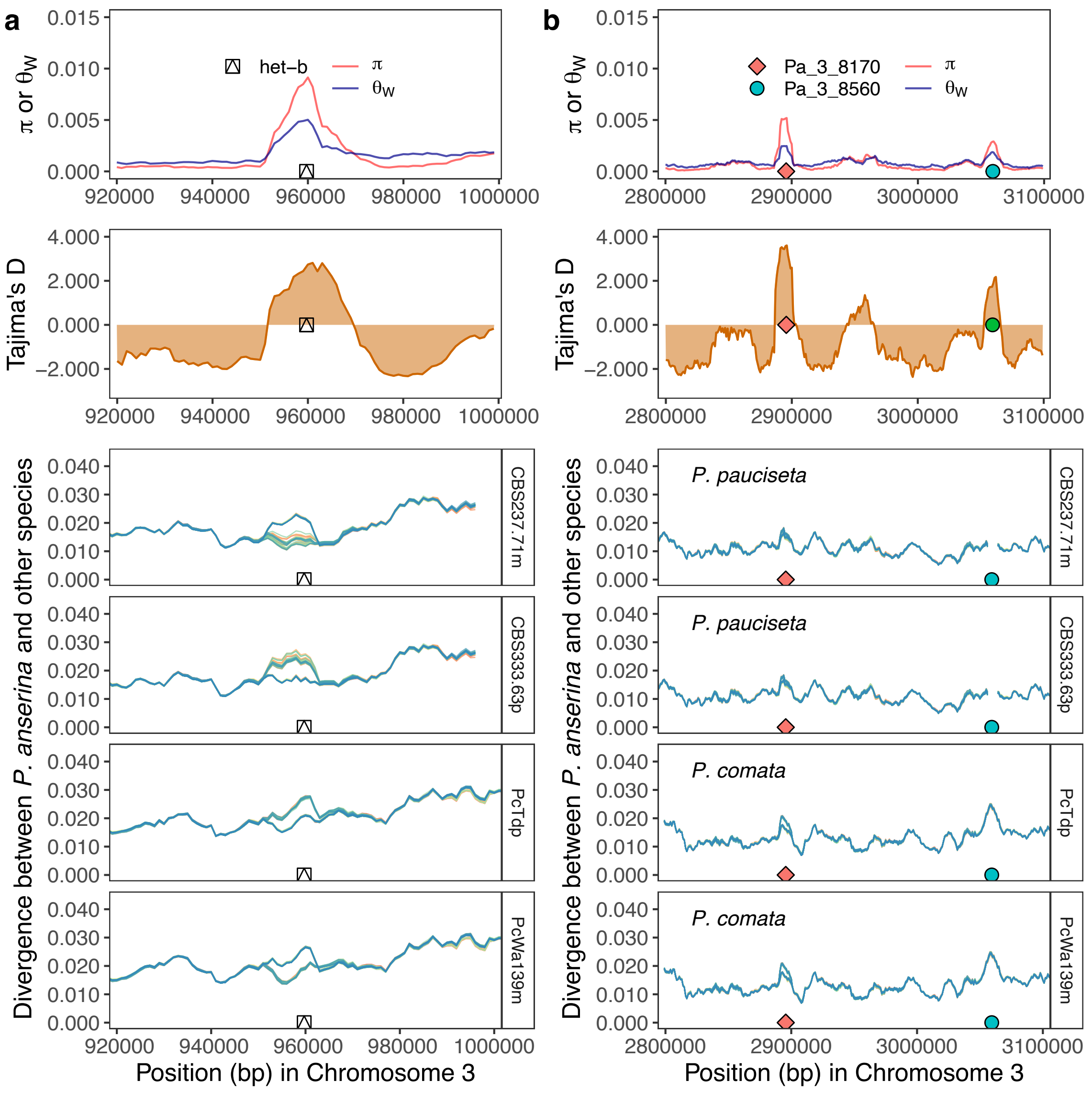

Figure S8. Population diversity and sequence divergence around *het-b* and two NLRs potentially evolving under balancing selection. The top panels correspond to the two metrics of genetic diversity π and Watterson's θ as well as Tajima's *D* in 10 kb windows (steps of 1 kb), as estimated in Ament-Velásquez et al. (2022) for the Wageningen population of *P. anserina*. The lower pannels compare the pairwise-divergence between all 106 *P. anserina* strains vs. two different strains of either *P. pauciseta* and *P. comata*. Each *P. anserina* strain line was assigned a different color. (a) Close-up to *het-b* region. (b) Close-up to *Pa_3_8170* and *Pa_3_8560* region. Notice that *Pa_3_8560* is absent in *P. pauciseta*; conversely in *P. comata*, *Pa_3_8560* was in fact translocated to chromosome 5, but the reads mapped to its homolog in the *P. anserina* reference assembly (chromosome 3).

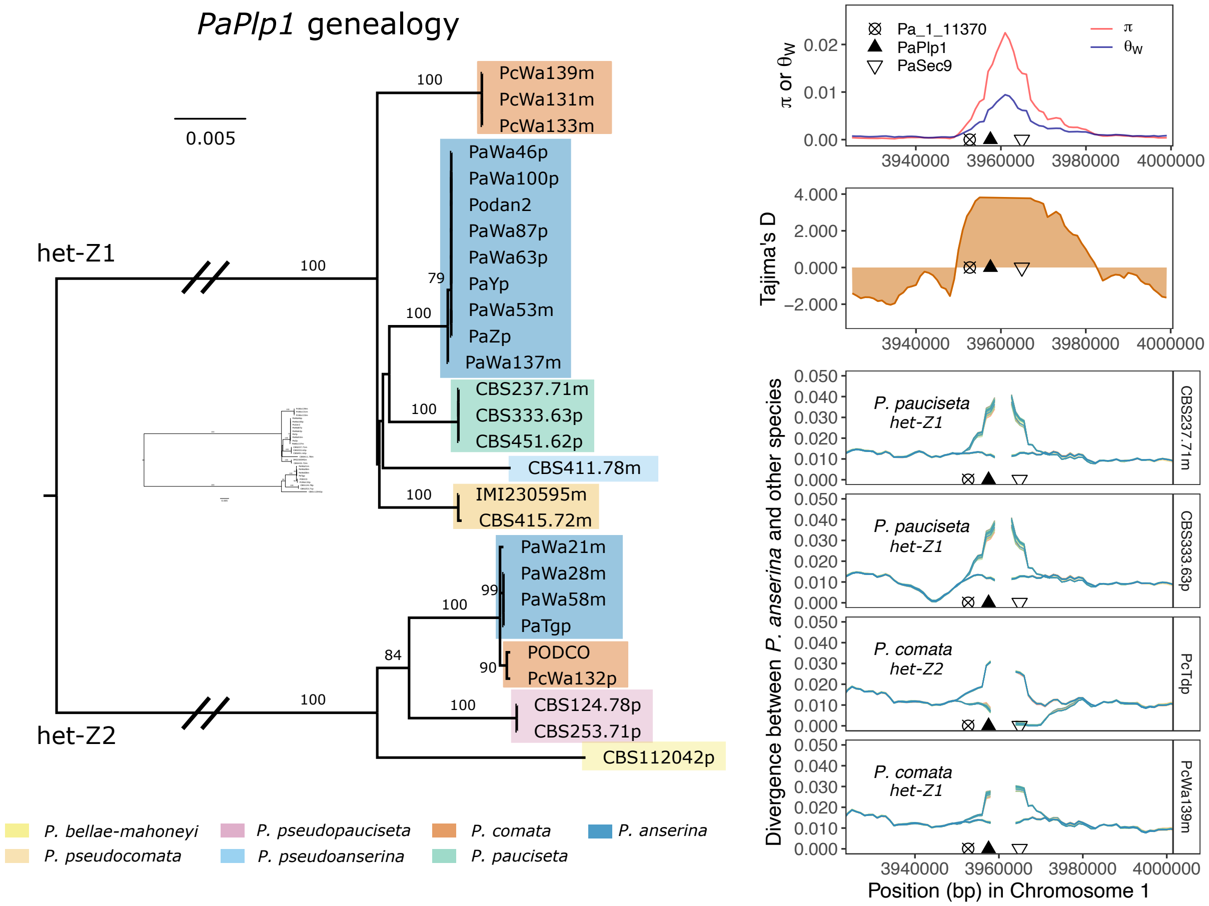

Figure S9. Balancing selection and introgression patterns at *het-z*. The *het-z* locus is composed of the NLR *PaPlp1* and its cognate *PaSec9*. To the left, a maximum likelihood phylogeny of the *PaPlp1* gene shows a clear division of the two *het-z* alleles. Branches are shown to scale as indicated by the scale bar (nucleotide substitutions per site). Long internal branches have been reduced for clarity, with a miniature version of the full tree in the middle. Branch support values correspond to standard non-parametric bootstrap (low within-species values are omitted for clarity). To the right, the top panels correspond to the two metrics of genetic diversity π and Watterson's θ as well as Tajima's *D* in 10 kb windows (steps of 1 kb), as estimated in Ament-Velásquez et al. (2022) for the Wageningen population of *P. anserina*. The lower panels compare the pairwise-divergence between all 106 *P. anserina* strains vs. two different strains of either *P. pauciseta* and *P. comata* (with their respective *het-z* alleles labeled). Each *P. anserina* strain line was assigned a different color (gaps correspond to missing data).

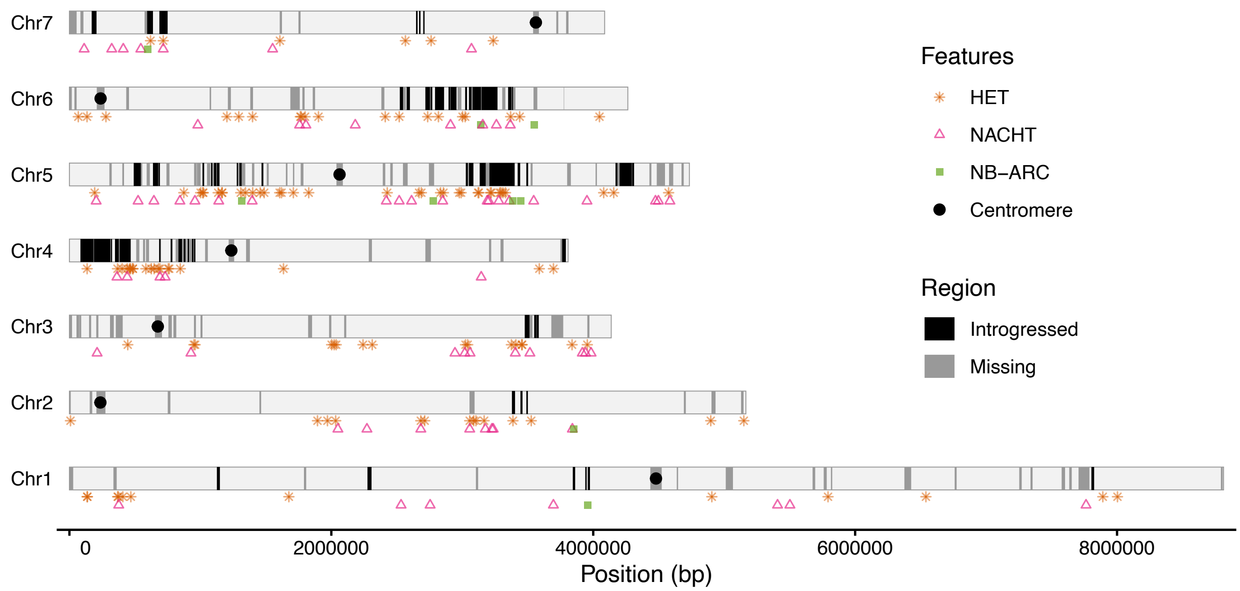

Figure S10. Chromosomal distribution of some NLR-associated domains (as annotated by InterProScan) in relation to introgressed regions from *P. pauciseta* or *P. comata*. The reference genome was divided into 10 kb-long overlapping windows (1 kb steps), which were classified as either introgressed (black) or regions with more than half missing data (gray).

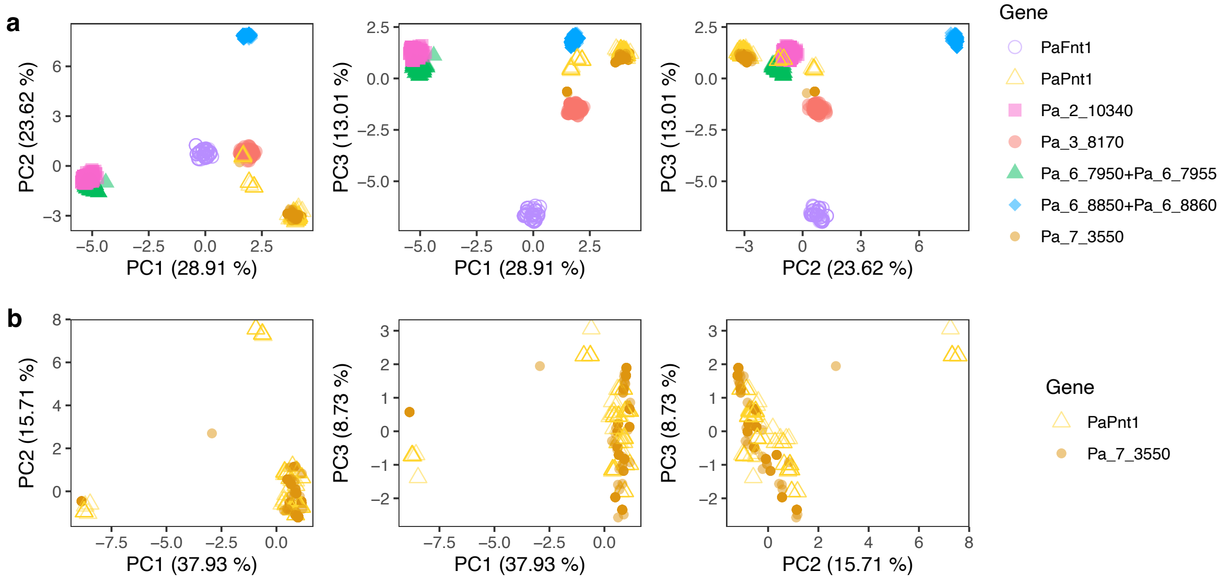

Figure S11. Principal component analysis of the TPR repeats with HIC. Considering all NLRs with HIC TPR results in strong differentiation for most genes (**a**). An exception is *PaPnt1* and *Pa_7_3550* which are largely overlapping (**b**). The genes *Pa_2_10340* and *Pa_6_7950+Pa_6_7955* are also overlapping but they are closely-related genes and hence expected to share similarities.

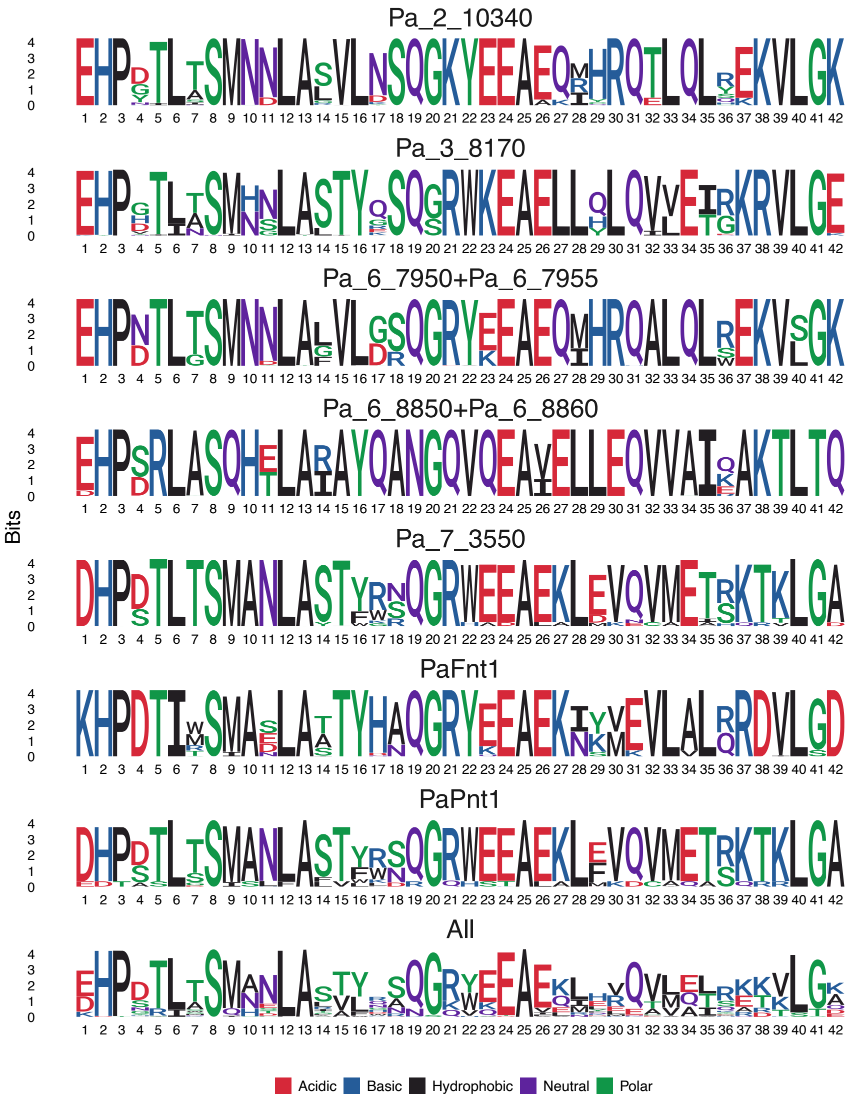

Figure S12. TPR logo of the NB-ARC genes with HIC in *Podospora anserina*.

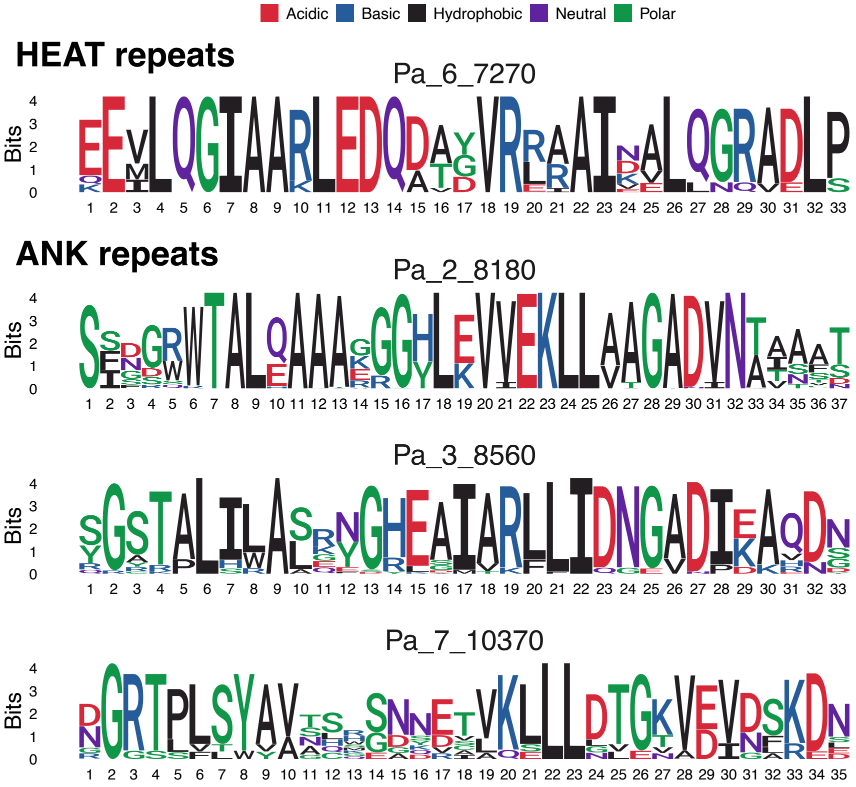

Figure S13. Logos of HEAT and ANK NLR genes with HIC in *Podospora anserina*.

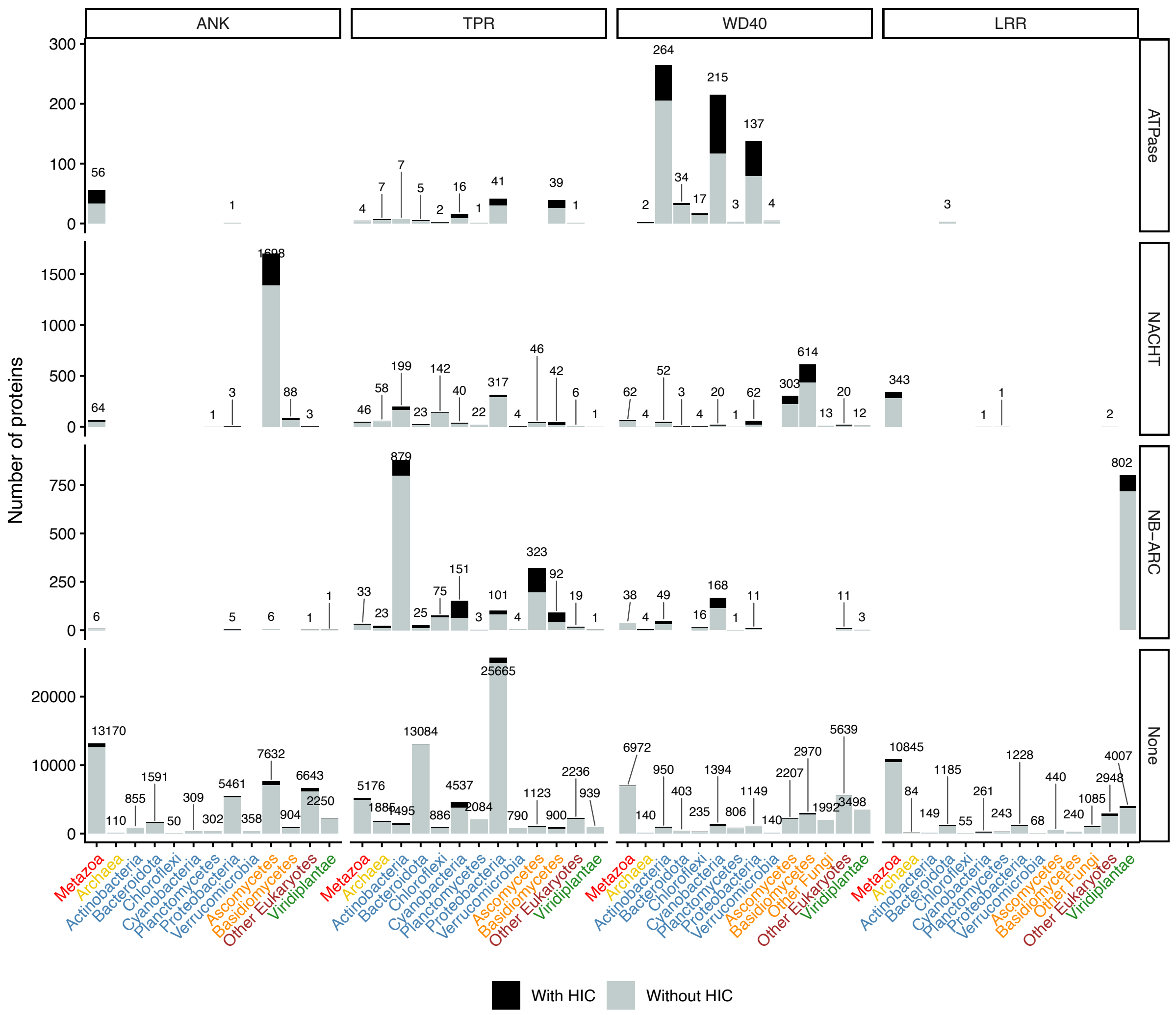

Figure S14. Abundance of superstructure-forming repeat proteins from the SMART dataset per taxonomic group and repeat type after reducing redundancy. The proportion of proteins with and without high internal conservation (HIC) is illustrated with gray and black colors. The "ATPase" category includes the nSTAND1 (PFAM code: PF20702), nSTAND2 (PF20703), and nSTAND3 (PF20720) domains. The NACHT category encompasses a number of related domains (PF13191, PF13238, PF13401, PF13479, PF13671, and PF24883). The NB-ARC category corresponds to the PFAM code PF00931. The "None" category are proteins that were not annotated with any of the above ATPase domains by InterProScan.

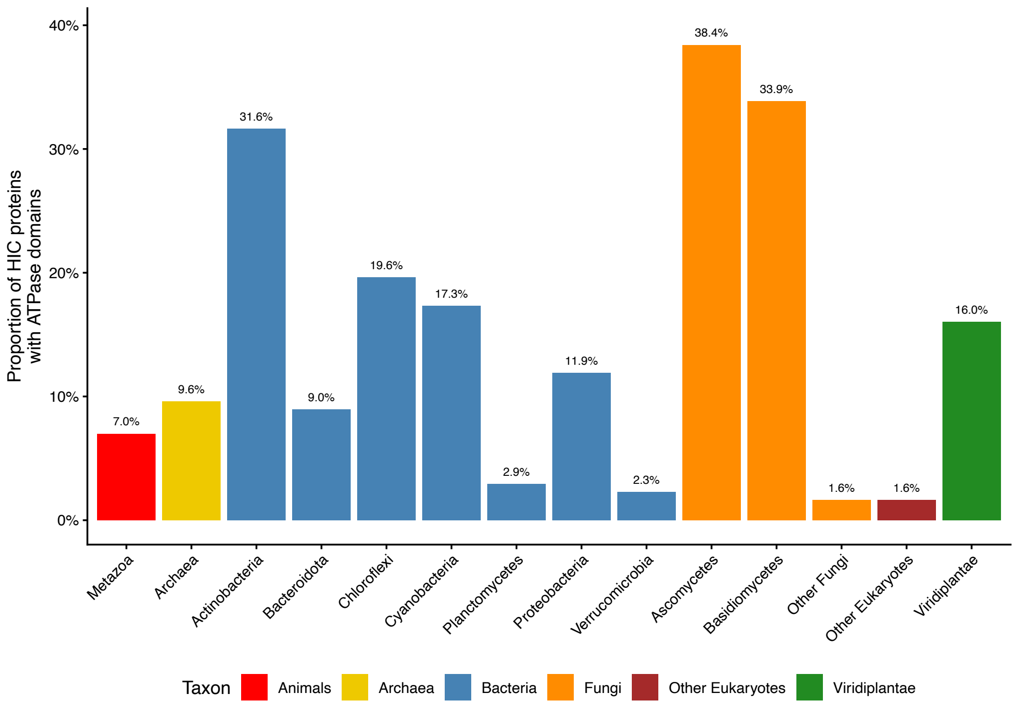

Figure S15. Proportion of superstructure-forming repeat proteins with High Internal Conservation (HIC) and ATPase domains across various taxonomic groups.
